# Initiation and rapid titration of methadone and slow-release oral morphine (SROM) in an acute care, inpatient setting: a case series

**DOI:** 10.1186/s40001-023-01538-0

**Published:** 2023-12-08

**Authors:** Laura Rodger, Maya Nader, Suzanne Turner, Erin Lurie

**Affiliations:** 1 Department of Medicine, University of Toronto, Toronto, Canada; 2 Department of Family & Community Medicine, University of Toronto, Toronto, Canada; 3 Department of Family Medicine, McMaster University, Hamilton, Canada; 4 Present Address: Wellesley St-James Town Health Centre, 95 Homewood Ave, Toronto, ON M4Y 1J4 Canada

**Keywords:** Opioid use disorder, Opioid agonist treatment, Methadone, Fentanyl, Injection drug use

## Abstract

**Background:**

Methadone titration in an outpatient setting typically involves initiation with subtherapeutic doses with slow titration to mitigate the risks of respiratory depression and overdose. In pregnancy, and generally, subtherapeutic doses of methadone and slow titrations are associated with poorer outcomes in terms of treatment retention and ongoing illicit opioid use. We aim to describe rapid titration of OAT in an inpatient setting for pregnant injection opioid users with high opioid tolerance secondary to a fentanyl-based illicit drug supply.

**Methods:**

Retrospective case series of patients admitted to a tertiary center with a primary indication of opioid withdrawal and treatment for severe opioid use disorder in pregnancy.

**Results:**

Twelve women received rapid methadone titrations with or without slow-release oral morphine for opioid use disorder during a total of fifteen hospital admissions. All women included in the study were active fentanyl users (12/12). Methadone dosing was increased rapidly with no adverse events with a median dose at day 7 of 65 mg (IQR 60–70 mg) and median discharge dose of 85 mg (IQR 70–92.5 mg) during their admission for titration. Slow-release oral morphine was used in half of the titration admissions (8/15) with a median dose of 340 mg (IQR 187.5–425 mg) at discharge. The median length of admission was 12 days (IQR 9.5–15).

**Conclusions:**

A rapid titration of methadone was completed in an inpatient setting with or without slow-release oral morphine, without adverse events showing feasibility of this protocol for a pregnant population in an inpatient setting. Patients achieved therapeutic doses of methadone (and/or SROM) faster than outpatient counterparts with no known adverse events.

## Background

The opioid crisis continues to be a significant public health concern globally with significant and increasing mortality risks [1–3]. This has increased with the prevalence of fentanyl as the opioid used by those with opioid use disorder (OUD) [4]. Opioid agonist treatment (OAT) has been shown to be safe and effective treatment for opioid use disorder, significantly reducing the mortality risk associated with illicit opioid use [5, 6]. The risk associated with titration of a full opioid agonist such as methadone in the first 30 days of titration has been the basis for titration protocols in many guidelines. Rapid and unpredictable accumulation leading to respiratory suppression, arrest and death are risks associated with the prolonged half-life, and varied metabolism of methadone, and therefore, care must be taken during titration [7]. However, much of the risk associated with early methadone titration is based on tolerance to lower potency opioids that were commonly used prior to the high prevalence of fentanyl [8–10]. Furthermore, North American expert consensus suggests adaptation of novel methadone induction protocols given the prevalence of fentanyl and fentanyl analogues in the drug supply [11, 12].

The newest form of OAT is slow-release oral morphine (SROM). SROM is an effective substitute for methadone or buprenorphine for those whom first line treatment with methadone and/or buprenorphine is not effective or for those with dose limiting issues, including QT prolongation [13]. Observed doses of once daily SROM provide effective management of opioid withdrawal and manage cravings [14, 15]. A previous case report utilizing SROM as an adjuvant to methadone in pregnancy demonstrated favorable pregnancy and neonatal outcomes [16]. Furthermore, recent guidelines specific to Canada suggest utilizing SROM as a bridge to support withdrawal management while working towards a therapeutic dose of methadone [12, 17].

Retention in treatment to an opioid agonist therapy reduce in mortality for those with opioid use disorder [18]. Treatment satisfaction, and higher methadone doses have played a role in predicting treatment retention for patients stabilizing on methadone maintenance treatment [19]. Given the higher potency of fentanyl to heroin, novel methods of initiation of methadone should be considered. Single case reports have shown safety of rapidly titrating methadone in an inpatient setting [16, 20–22]. Furthermore, institutional guidelines have also been developed for more rapid initiation [23]. As these cases and protocol have shown, rapid induction can be facilitated in an inpatient setting, reducing the time to therapeutic dosing when compared with restrictive, outpatient titration schedules.

Pregnancy presents a unique circumstance, where the titration of OAT and management of opioid withdrawal are considered a primary indication for hospital admission due to risk to the pregnancy and fetus [24, 25]. In pregnancy, subtherapeutic doses of methadone and slow titrations are associated with poorer outcomes in terms of retreatment retention and ongoing illicit opioid use [26, 27]. We aim to describe rapid titration of OAT in an inpatient setting for pregnant injection opioid users with high opioid tolerance secondary to a fentanyl-based illicit drug supply [28]. This study aims to describe the safety and feasibility of rapid OAT induction within this population.

## Methods

This case series aims to describe patients who had rapid methadone titration with and without the addition of slow-release oral morphine (SROM) titration in an inpatient setting, where the primary indication for admission was rapid titration in the context of pregnancy. All Strengthening the Reporting of Observational Studies in Epidemiology (STROBE) [29] reporting guidelines for cohort studies were incorporated into reporting of this case series. Ethics approval was granted by the St. Michael’s Hospital Research Ethics Board (REB #20-093).

### Setting

St. Michael’s Hospital is a large, tertiary care institution located in some of Toronto’s lowest income postal codes [30]. This hospital serves a large volume of patients who use opioids, with recent non-published hospital data showing a higher proportion of opiate overdoses triaged to its Emergency Department compared to other downtown tertiary centers (unpublished data).There are high rates of patients with serious mental health, addictions, and homelessness in this geographic area. The consultation, inpatient perinatal addictions team can admit pregnant patients for stabilization of OUD with OAT at any gestation on the antepartum unit at the hospital. The patients are followed by addiction medicine as well as a primary care obstetrics team with expertise in substance use in pregnancy.

### Inclusion criteria

Inclusion criteria consisted of adult patients (> 18 years of age) with opioid use disorder admitted to hospital during their peripartum period for opioid withdrawal and titration of opioid agonist therapy (OAT). Cases were included if there was an admission to the antenatal unit for the purpose of initiating opioid agonist treatment as a new start or to restart opioid agonist treatment (after missed dosing necessitating a new start of OAT). Treatment refers specifically to methadone with or without the addition of sustained release oral morphine (SROM) or immediate release (IR) morphine. Admissions between January 1, 2016 and August 1st, 2020 were included in the study.

### Exclusion criteria

Predetermined exclusion criteria were admission for less than or equal to 3 days, which would not permit significant titration or monitoring. Notably, two cases were excluded, to avoid duplication, as they have been reported and published separately. Admissions for buprenorphine titration or rotation were also excluded.

### Data collection and statistical analysis

Demographic and clinical variables were obtained from the electronic and paper medical records from St. Michael’s Hospital. Cases were still included if demographic or clinical variables were missing; however, all cases had complete medication administration records (MAR). Data collected included age at admission, gestational age at admission, sexually transmitted infections, bloodborne viral infections (HIV status and hepatitis C [HCV] status). Details regarding injection drug use were self-reported (substance, amount, and route) and compared to urine drug screen (UDS) results when available.

The MAR was used to determine the total daily dose of opioid agonist therapy (methadone and/or SROM) in addition to the total daily dose of opioids (including both scheduled and as needed (PRN) medications). Opioid agonist therapy is reported as the total daily dose but was administered with split dosing in some cases. Where available, the dose of OAT at the time of presentation to labor and delivery was recorded as an indicator of retention in treatment between titration and delivery (if admitted on a similar dose of OAT) or not retained in treatment (not currently receiving OAT at the time of admission for labor). A morphine equivalent of 8:1 methadone to morphine, as has previously been published and utilized in other methadone studies, was used for calculation purposes [31].

Categorical variables are presented as frequencies and percentages. Continuous variables are presented as median with interquartile range (IQR) as the data are not normally distributed and from a small sample. Descriptive statistics were performed using Excel 2019.

## Protocol

**Fig. 1:**
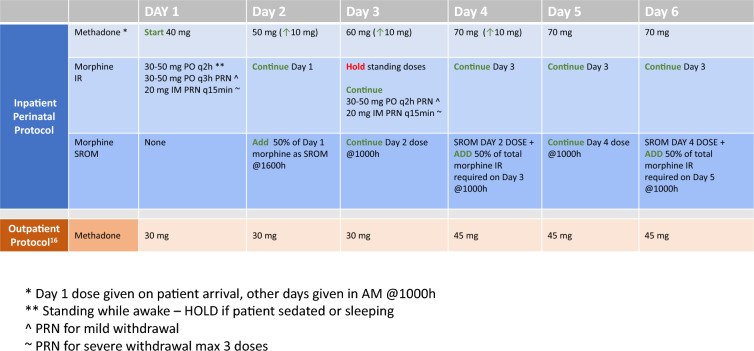
Demographics

The rapid methadone titration protocol was based on the inpatient methadone titration guidelines from the University of California San Francisco. The majority of patients were started on methadone 30 mg and titrated by 10 mg daily to a maximum of 70 mg at which time this dose was held for 3 days to reach steady state. After that time patients were offered a maximum of a 15 mg increase and held at this dose for a minimum of 5 days before another methadone dose was undertaken. When SROM was added an initial dose of 100 mg was added approximately 8 h after methadone to avoid the peak effect of methadone. Given the high tolerance of our patient population, and to avoid withdrawal which may lead to obstetrical complications, standing doses of immediate release (IR) morphine 30–50 mg every 2 h were provided with equivalent as needed doses available for pain, withdrawal, or cravings. Standing doses had the caveat to not be administered if patients showed signed of oversedation or were sleeping. Approximately every 2 days, 50% of the total morphine IR was converted to SROM and the standing dose of morphine was reduced until the patient was only on SROM (Fig. 1).

## Results

Twenty-four admissions for opioid withdrawal were identified. Four patients had two titration admissions within the same pregnancy (one titration was excluded as the length of stay in hospital was 3 days). An additional admission was excluded as consultation was for continuation of methadone during antenatal hospitalization, and no titration in hospital was required. Other excluded admissions were postpartum titration (*n* = 2) buprenorphine inductions (*n* = 1) or scheduled opioid rotations to buprenorphine and oral morphine (*n* = 2). In total, 15 titration admissions were considered in 12 patients.

The mean age at admission was 31 (± 5.1 years). Demographic data are summarized in Table 1. Most patients reported polysubstance use (75%, 9/12), with crack-cocaine and/or methamphetamine use reported with opioid use in all cases. The self-reported opioid use and/or urine toxicology confirmed fentanyl as the opioid of choice in all patients. All patients used fentanyl via injection. Nicotine use was also common, with 83% of women reporting cigarette smoking (10/12). There were no women who reported concurrent alcohol use. Most admissions were during the third trimester (73%, 11/15). There were no admission in the first trimester, four admissions in the second trimester (27%, 4/15).

**Table 1:** Demographics of patients initiated on OAT in hospital

	*N* = 12 (*n*, %)	Median (IQR)
Age		30.5 (27–33.75)
Single	5 (42)	
No fixed address	6 (50)	
Income		
Social assistance	7 (58)	
Employment	1 (0.83)	
Unknown	4 (33)	
Fentanyl use reported	12 (100)	
Fentanyl on UDS*	6	
Injection use	12 (100)	
Prior OAT**	6 (50)	
HIV	0	
HCV	7 (58)	

^*^Only 6 UDS results available

^**^ Ever previously receiving OAT

All patients remained in hospital for the duration of their opioid agonist titration. Half of the women were starting OAT for the first time, with 6 women reporting previously receiving OAT (50%, 6/12). The median length of stay was 12 days (IQR 9.5–15 days). The median daily dose of methadone at discharge was 85 mg (IQR 70–92.5 mg), equal to 680 mg morphine equivalents (MEq). SROM was used as an adjunct for 9 titrations (60%, 9/15) and the median dose of SROM at discharge was 340 mg (187.5–425). For women that had combined methadone and SROM, the median combined MEq was 1020 mg. Overall, women rarely missed doses of methadone in hospital (*n* = 2 titrations, each with a single missed dose when the patient was not present in the room). There were no significant adverse events, including no sedation, respiratory suppression, or opioid overdose (Table 2).

**Table 2:** Doses and characteristics of OAT received by women who were initiated on rapid titrations

	Median (IQR)	*N* = 15 titrations (n, %)
Methadone (mg)		
Day 1	30 (30–42.5)	15 (100)
Day 4	50 (42.5–60)	15 (100)
Day 8	70 (65–75)	15 (100)
Discharge dose	85 (70–92.5)	15 (100)
Slow-release oral morphine (mg)		
Day 1	110 (105–115)	2 (12.5)
Day 4	130 (87.5–200)	8 (50)
Day 8	260 (200–300)	8 (50)
Discharge dose	340 (187.5–425)	8 (50)
Morphine Equivalents** (mg)		
Day 1	37.5 (31.25–133.75)	15 (100)
Day 4	150 (46.5–367.5)	15 (100)
Day 8	175 (90–332.5)	15 (100)
Discharge dose	*Not applicable*	*Not applicable*
Hospital adverse events		0
Planned discharges		15/15
Patients seen in follow-up		9/12
Maintained OAT until delivery		5/11*(45)

^*^Exclusion of incarcerated individual

^**^Including n = 1 case prescribed PRN hydromorphone

Post-discharge from titration, 9/12 (75%) women were followed on an outpatient setting within a week of discharge. Of the 3 who did not follow-up as an outpatient after methadone titration, one of these women was incarcerated, one had delivered during admission and did not have postpartum follow-up, the last was lost to follow-up. Of the 9 women who had ongoing follow-up on an outpatient basis, none had adverse effects of their OAT, including sedation, or respiratory depression post-discharge.

## Discussion

We describe a group of patients, highly tolerant to opioids and actively using illicit fentanyl, who were hospitalized for rapid methadone (± SROM) titration. Admissions to hospital for primary OAT titration are available only to pregnant patients in our geographical area. Therefore, this is a unique population, where admissions for primary OAT titration in an inpatient setting are possible. The population identified in this study had evidence of psychosocial instability: over 50% were homeless, almost half were not partnered, and over 90% were unemployed and/or on social assistance. All women were actively using fentanyl via injection. To our knowledge, this represents the first case series of patients using fentanyl in pregnancy in which OAT was rapidly titrated, complementing a recent case study showing feasibility of this method of titration [21].

There is an emerging concern that current methadone guidelines internationally are inadequate to retain patients in care and reduce their illicit substance use, especially in light of the increase in highly potent fentanyl analogues [32]. It is also felt that patients who use fentanyl have high opioid tolerance, necessitating higher doses of methadone [33]. Fentanyl is associated with an increase in overdose deaths, and higher doses of methadone may be more protective against overdose death [34]. Furthermore, higher doses of methadone (considered to be > 80 mg/day) have been associated with reduced illicit drug use in non-fentanyl using pregnant populations [35]. In pregnancy, doses over 60 mg of methadone are associated with better treatment retention and reduction in illicit substance use [36]; however, these data are not available for primarily fentanyl-using populations. It is reasonable to assume that higher doses may be required in fentanyl-using populations. Therefore, there is an urgent need for protocols that will allow rapid titration of methadone and full-opioid agonists that will stabilize patients on methadone equivalents in the 60–80 mg range as a minimum. Our protocol demonstrates that in an inpatient-setting we are able to exceed or meet this target more rapidly than as an typical outpatient titration would allow.

Guidelines, both national and international, have similar recommendations for initial dosing of methadone for opioid use disorder. Dose initiation in the WHO guidelines is suggested to be much lower than our local guidelines, with starting doses of less than 20 mg recommended [37]. Local protocols for methadone titration in Canada, suggest an outpatient starting doses of 10–30 mg with subsequent dose increases up to 10 mg every 3–5 days [17]. Locally, the fastest titration to a dose of 80 mg of methadone requires a minimum of 15 days. It is often also difficult to get patients to a therapeutic dose of methadone due to limitations in outpatient protocols, including missed doses, and missed appointments for titration. As such, many patients are maintained on subtherapeutic doses below 60–100 mg, which impacts continuation of OAT [37]. Previous cases reported by our group have highlighted novel approaches to OAT, where methadone and SROM were rapidly titrated [16, 20–22]. These rapid initiation and titrations have been reported in a non-pregnant patient as well as in pregnancy. Like these cases, there were no documented adverse events within this cohort of rapid titrations based on review of the medical charts, including no doses withheld secondary to sedation, respiratory suppression or overdose, and no in-hospital complications. All women remained in hospital for at least 7 days.

Treatment retention is challenging with patients with severe opioid use disorder. In non-pregnant patients, younger age, specific substance used (cocaine and heroin), lower doses of methadone, criminal activity or incarceration, and negative attitudes towards methadone are associated with reduced retention [38]. Overall, methadone is associated with longer treatment retention and fewer relapses when compared to nonpharmacologic therapy, especially at doses greater than 60–80 mg daily [19, 37, 39, 40]. Rates of discontinuation of OAT [41] in pregnancy are generally cited to be in the 0–33% range and are higher in the post-partum period [40]. In this study, discontinuation, as measured by the number of patients initiated on OAT in hospital and presenting in labour on OAT, was high in our patient population. Further research is needed to determine whether (a) patients using fentanyl have higher discontinuation rates (particularly on doses < 80 mg of methadone) and (b) whether patients who inject drugs may have higher discontinuation rates as well. It is possible that these two factors play into the discontinuation rates seen in this study, but also the lack of housing, minimal financial and social supports may also play into the rates of discontinuation.

This is a descriptive study which describes a small number of cases, adding to three cases previously published in pregnancy [16, 20, 21] and one case in a non-pregnant individual [22]. Limitations of this study include important pharmacokinetic considerations in pregnancy, with the physiologic changes in pregnancy impacting methadone dosing [42, 43]. Specifically, methadone clearance increases in the third trimester which can cause withdrawal symptoms necessitating a dose increases as the pregnancy continues [42]. In addition, we were not able to comment on details of prior OAT initiations, reasons for previous unsuccessful titrations or circumstances affecting stabilization or retention which are relevant to any OAT start. The generalizability is low, given the availability of on-demand beds specifically for the purpose of rapid methadone titrations are rare and highly experienced inpatient addiction medicine clinicians are not available routinely in acute care hospital. Only short-term follow-up information was available, and although we saw no known adverse events, we cannot rule out the potential for harm; although, the similar work described previously has also not demonstrated adverse events. We acknowledge that the treatment in these cases was not in keeping with current guidelines locally or internationally. Literature guiding adjunctive use of SROM is limited to case reports, although it is now accepted as a standalone agent for OAT. Availability of SROM is limited in some settings [41]. However, given the increasingly toxic illicit opioid supply and associated morbidity and mortality, trialing aggressive and novel treatment approaches in a monitored, acute care setting is warranted. There is no proposed protocol of how to titrate unstable pregnant women who are actively using fentanyl effectively on opioid agonist therapy.

## Conclusion

This is the first case series to describe a novel and inpatient protocol for rapid titration of methadone using SROM as an adjuvant to achieve high morphine equivalents (and, therefore, evidence-based therapeutic doses) to prevent withdrawal and cravings in highly tolerant, pregnant patients with severe OUD. We provide additional preliminary evidence that rapid methadone titration (± SROM) can be accomplished in a monitored setting without sedation, respiratory depression, or overdose. We advocate for inpatient monitoring in rapid titrations, or close outpatient follow-up in line with the previous case report of rapid titration [21, 22]. Further research is needed to review the safety of rapid outpatient titrations and what criteria would be needed to consider outpatient titrations.

## Data Availability

The data set used during the current study are available from the corresponding author on reasonable request.

## Abbreviations

OUD: Opioid use disorder
OAT: Opioid agonist therapy
SROM: Slow-release oral morphine
HCV: Hepatitis C virus
HIV: Human immunodeficiency virus
MEq: Morphine equivalents
